# Point-of-Care Diagnostic Test for Rapid Detection of Infectious Laryngotracheitis Virus by Loop-Mediated Isothermal Amplification and Nanoprobes

**DOI:** 10.3390/ijms26051971

**Published:** 2025-02-25

**Authors:** Pablo Cea-Callejo, Claudia Trenado, Elías El Mansouri, Esperanza Gomez-Lucia, Ana Doménech, Mar Biarnés, J. Marco Cuenca, Christian J. Sánchez-Llatas, Ricardo Madrid, Laura Benítez

**Affiliations:** 1 Department of Genetics, Physiology, and Microbiology, School of Biology, Complutense University of Madrid (UCM), 28040 Madrid, Spain; pcea@ucm.es (P.C.-C.); clautren@ucm.es (C.T.); eliaselm@ucm.es (E.E.M.); chsanc01@ucm.es (C.J.S.-L.); 2 Research Group of “Animal Viruses”, Complutense University of Madrid, 28040 Madrid, Spain; duato@ucm.es (E.G.-L.); domenech@vet.ucm.es (A.D.); 3 Department of Animal Health, Veterinary Faculty, Complutense University of Madrid (UCM), 28040 Madrid, Spain; 4 Centro de Sanidad Avícola de Cataluña y Aragón (CESAC), 43206 Reus, Spain; mbiarnes@cesac.net; 5 Department of Physical Chemistry, School of Chemistry, Complutense University of Madrid (UCM), 28040 Madrid, Spain; juliocue@ucm.es

**Keywords:** infectious laryngotracheitis virus (ILTV), molecular detection, loop-mediated isothermal amplification (LAMP), nanoprobes, point-of-care test (POCT)

## Abstract

Infectious laryngotracheitis virus (ILTV), a DNA virus classified as *Gallid alphaherpesvirus 1*, causes a highly contagious respiratory disease in chickens, leading to significant economic losses and health risks for the poultry industry. The rapid detection of ILTV is essential to control its spread and prevent outbreaks. Traditional diagnostic methods like PCR are costly, require specialized personnel, and delay response efforts. To address this, we developed a point-of-care diagnostic test combining loop-mediated isothermal amplification (LAMP) with DNA nanoprobes on respiratory swabs. LAMP targets the ILTV-glycoprotein E (gE) gene, enabling rapid nucleic acid amplification at 65 °C without extraction, making it suitable for on-site detection. DNA nanoprobes provide a colorimetric readout visible to the naked eye. Gold nanoparticles drive this readout, as their red color, based on localized surface plasmon resonance, persists in the presence of ILTV DNA through DNA-DNA hybridization, ensuring reliable detection. The assay achieved 100% sensitivity and specificity for ILTV-gE, with a detection limit of 200 copies per reaction, allowing for the early identification of infections. The results are available within 45 min, enabling prompt measures to control ILTV spread. Cost-effective and user-friendly, this method enhances disease management and biosecurity in poultry farms.

## 1. Introduction

Avian infectious laryngotracheitis (ILT) is a highly contagious respiratory disease in chickens, which poses a considerable economic threat to the poultry industry worldwide, due to severe respiratory outbreaks. Depending on strain virulence, these cause high morbidity (90–100%) and variable mortality [1,2,3]. It is caused by infectious laryngotracheitis virus (ILTV), a herpesvirus classified as *Iltovirus gallidalpha 1* of the subfamily *Alphaherpesvirinae*, with a DNA genome of around 150 kb encoding 80 predicted viral protein open reading frames (ORFs). The disease usually occurs in acute or subacute forms with mortality rates ranging from 50% to 30%, respectively, with clinical respiratory signs including conjunctivitis and nasal discharge, a decrease in egg production, and weight loss. Its clinical signs resemble those caused by other respiratory viruses, such as infectious bronchitis virus (IBV) and avian metapneumovirus (aMPV). In addition, the existence of asymptomatic carriers makes virus contention more difficult. In severe forms of disease, characteristic hemorrhagic tracheitis is present [1,3,4]. ILTV establishes lifelong latency in the trigeminal ganglia, and birds become long-term carriers, complicating disease control on farms [1,2,5].

ILTV transmission occurs easily in infected chickens, mainly through close contact. This is rapidly enhanced by intensive farming. It is crucial to rapidly detect the virus to implement the most appropriate measures and reduce its economic impact on production. Although conventional laboratory methods remain useful, they are time-consuming and relatively expensive, so they have been replaced by conventional PCR and real-time PCR (qPCR) [6,7]. Rapid and simple on-farm application of these molecular techniques is currently hindered by numerous limitations. Thus, there is growing interest in developing point-of-care tests (POCTs) for on-farm diagnostics to mitigate outbreaks and enhance animal welfare. Ideally, POCTs should be affordable, sensitive, specific, user-friendly, rapid, and robust, requiring only minimal equipment [8,9].

The loop-mediated isothermal amplification (LAMP) technique offers several advantages over PCR, making it an ideal technique for POCTs. Among these, the ability of LAMP to perform amplifications at a constant temperature at 60–72 °C for less than 1 h stands out [10]. In addition, the *Bacillus stearothermophilus* (Bst) enzyme’s strand displacement activity eliminates the need for denaturation, simplifying amplification and obviating the need for complex thermal cycling and expensive thermocyclers. Moreover, this enzyme shows remarkable robustness and resistance to inhibitors, making LAMP effective for analyzing a variety of clinical samples, with minimal pre-processing requirements in field conditions [11]. Consequently, developing a POCT-compatible lysis buffer is essential to enable LAMP deployment in resource-limiting settings. The reaction takes place using two primer pairs: the internal primers (FIP-BIP), which are added in a higher concentration so that they bind first to the target, and the external primers (F3-B3), which are present in the reaction at a lower concentration. Each inner primer is designed to target the original and the complementary strand, allowing for the formation of a loop during amplification. The efficiency of the reaction can be enhanced by adding a loop primer pair (LF-LB). The use of four up to six primers to target specific regions makes design more complex than for PCR. However, this complexity yields higher sensitivity and specificity, as LAMP can distinguish eight specific DNA sequences, whereas PCR targets only two [9,12]. The exponential amplification enables rapid results, and POCT-compatible visualization techniques are well-established. Because of these characteristics, LAMP has been successfully applied to the detection of several respiratory viruses in chickens [5], including the detection of ILTV [13,14,15].

Among the POCT-compatible visualization techniques for LAMP, we find the use of pH indicators, metal indicators (turbidity-based methods), or colorimetric or fluorescent dyes as intercalating agents [5]. These methods, however, lack target specificity. The use of gold nanoparticles (AuNPs), with their localized surface plasmon resonance (LSPR) properties, has emerged as a promising approach to enhance LAMP-based techniques. Typically observed as a distinctive red color around 520 nm and visible to the naked eye, this phenomenon is dependent on the size, shape, and composition of the AuNPs. Consequently, the SPR angle produced on the reflected light wave is specific to a determined surface of the AuNPs [14]. When functionalized with specific oligonucleotides, as DNA nanoprobes, a precise, sensitive, and accurate visualization system for enriched LAMP products is achieved. For instance, under high-salt conditions and in the absence of the specific LAMP product, the red color of DNA nanoprobes changes; it either shifts to longer wavelengths or clears completely, becoming visible to the naked eye. In contrast, the presence of the target LAMP product promotes DNA-DNA hybridization, preserving the red color [16].

This study aimed to validate a highly specific, rapid, affordable, and user-friendly ILTV detection system. This LAMP-based assay, combined with specific DNA nanoprobes targeting the ILTV-gE gene, enables rapid, visual, and in situ ILTV diagnosis, helping to prevent its spread and minimizing associated economic losses on poultry farms.

## 2. Results

### 2.1. Optimization of LAMP Assay Targets and Conditions

**Figure 1:**
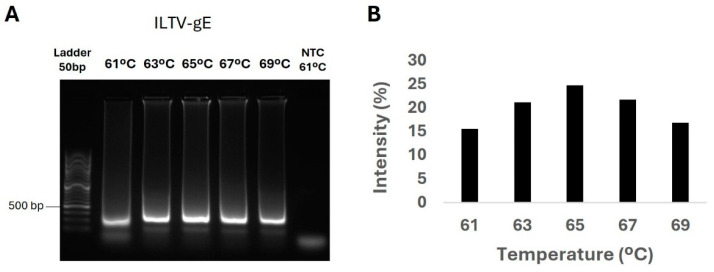
Optimization of LAMP reaction temperature for ILTV-gE. (**A**) Representative DNA electrophoresis of end-point LAMP products after a 30-min reaction at various reaction temperatures (61 °C to 69 °C) using the ILTV-gE LAMP primers. The shortest amplification size is 201 bp. (**B**) Histograms showing the densitometric quantitation of DNA electrophoresis indicating the percentage of the amplification product band (FIJI software, v2.16, accessed on 10 January 2025; https://fiji.sc); DNA extracted from the ILTV vaccine strain was used as a positive sample. NTC, non-template control (negative control).

The three sets of primers, targeting either the ILTV glycoprotein-E (ILTV-gE) or the ILTV thymidine kinase (ILTV-TK) genes, were tested under identical LAMP reaction conditions. Initial validation was performed detecting the ILTV Salisbury 146 vaccine strain as the positive control and a non-template control as the negative control. To optimize the LAMP reaction temperature for each primer set, a temperature gradient from 61 to 69 °C (at 2 °C intervals) was tested. Densitometric analysis of the amplification products (Figure 1) revealed that temperatures between 63 and 67 °C yielded the strongest signals for the gE target. Based on these results, 65 °C was selected as the optimal reaction temperature. 

### 2.2. LAMP Assays for Sample Detection

**Figure 2:**
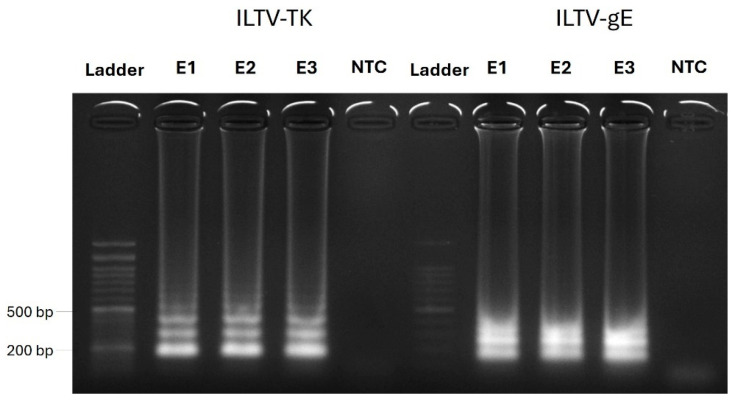
Representative DNA electrophoresis of end-point LAMP products generated using different primer sets for ILTV-TK and ILTV-gE at 65 °C during a 30-min reaction. The shortest amplification size is 219 bp for the ILTV-TK primer set and 201 bp for the ILTV-gE primer set. E1 to E3 correspond to three DNA/RNA extracts of ILTV-positive samples. NTC, non-template control (negative control).

Following successful optimization of both gE and TK LAMP amplification, a panel of 32 previously qPCR-validated DNA/RNA extracts (E1 to E32), including 22 ILTV-positive and 10 ILTV-negative samples, was analyzed. Samples were tested in individual reactions using either the ILTV-gE or ILTV-TK primer sets. The ILTV-gE LAMP assay achieved a sensitivity of 100% (32/32), while the ILTV-TK LAMP assay exhibited a lower sensitivity, approximately 84% (21/25) after a 30 min reaction time. Notably, both LAMP assays exhibited a specificity of 100% (10/10) (Figure 2). Based on these results, the ILTV-gE primer set was selected for further validation of the diagnostic system for ILTV with crude samples.

### 2.3. Quantitative LAMP (qLAMP) Assay with Crude Samples

**Figure 3:**
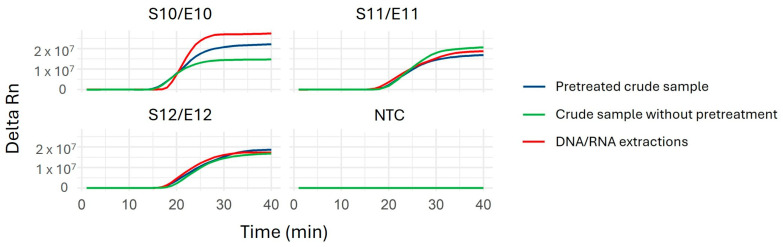
Optimization of the qLAMP protocol using three ILTV-positive samples: crude samples without pretreatment (S10–S12) and their corresponding DNA/RNA extracts (E10–E12), tested using the ILTV-gE primers. Representative amplification plots are shown for: total nucleic acid extractions (E samples, shown in red) and crude samples (S samples) treated with lysis buffer (blue) and untreated (green). Amplification times ranged from 15.1–17.2 min for sample S10/E10, 16.9–17.5 min for S11/E11, and 15.8–18.8 min for S12/E12. Additionally, a non-template control (NTC) was included for comparison alongside these three ILTV-positive samples.

To evaluate the performance of the LAMP assay, a panel of 47 crude samples (S1–S47) and their corresponding nucleic acid extractions (E1–E25) were analyzed. Initially, LAMP assays were carried out using crude samples pretreated with a rapid lysis buffer developed in our laboratory for the detection of enveloped viruses, as described in Section 4. Crude samples were tested with and without a rapid lysis buffer pretreatment designed for virus detection. As shown in Figure 3, the lysis buffer pretreatment did not significantly affect the detection time or efficiency, with comparable results observed between treated crude samples, untreated crude samples, and extracted DNA/RNA.

**Figure 4:**
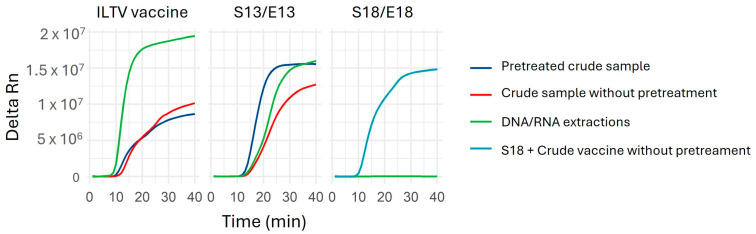
Optimization of qLAMP using ILTV vaccine, ILTV-positive sample (S13 and E13), and ILTV-negative sample (S18 and E18) using ILTV-gE primers. Amplification results are shown for DNA/RNA extractions (green), crude samples (S13 and S18) treated with lysis buffer (dark blue), and untreated (red). The S18 sample and the untreated ILTV vaccine were combined in a 1:1 ratio (light blue). Amplification times ranged from 8.7–10.1 min for the ILTV vaccine and 12.3–14.1 min for sample S13/E13.

The effect of freeze–thaw cycles on crude sample lysis was investigated using the live attenuated ILTV Salisbury 146 vaccine strain. Duplicate qLAMP assays were performed on the ILTV vaccine and ILTV-positive crude samples, comparing lysis buffer pretreatment, untreated samples, and total nucleic acid extractions. Additionally, to simulate real field conditions, a crude sample negative for respiratory viruses (sample S18) was spiked with the untreated ILTV vaccine strain to assess the efficacy of the lysis buffer pretreatment and potential LAMP inhibition. All samples were amplified successfully within a comparable timeframe, with no significant differences observed between preparation methods (Figure 4).

**Figure 5:**
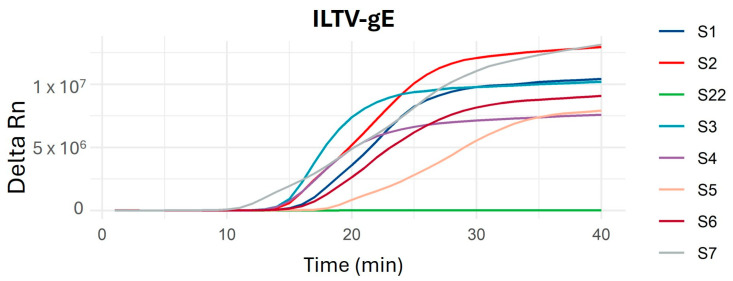
Representative qLAMP amplification plots using ILTV-gE LAMP primers and DNA/RNA extractions from ILTV-positive samples (S1 to S7). S22 was used as negative sample control; this sample was negative for ILTV and positive for aMPV.

Finally, the entire panel of crude samples (S1 to S47) was analyzed without any treatment. All positive samples previously identified by end-point LAMP using their corresponding DNA/RNA extracts (E1 to E25) were also successfully detected in their corresponding crude samples (S1 to S25), maintaining a 100% sensitivity with no false positives (Figure 5).

### 2.4. Validation of DNA Nanoprobe Detection System Coupled to LAMP

**Figure 6:**
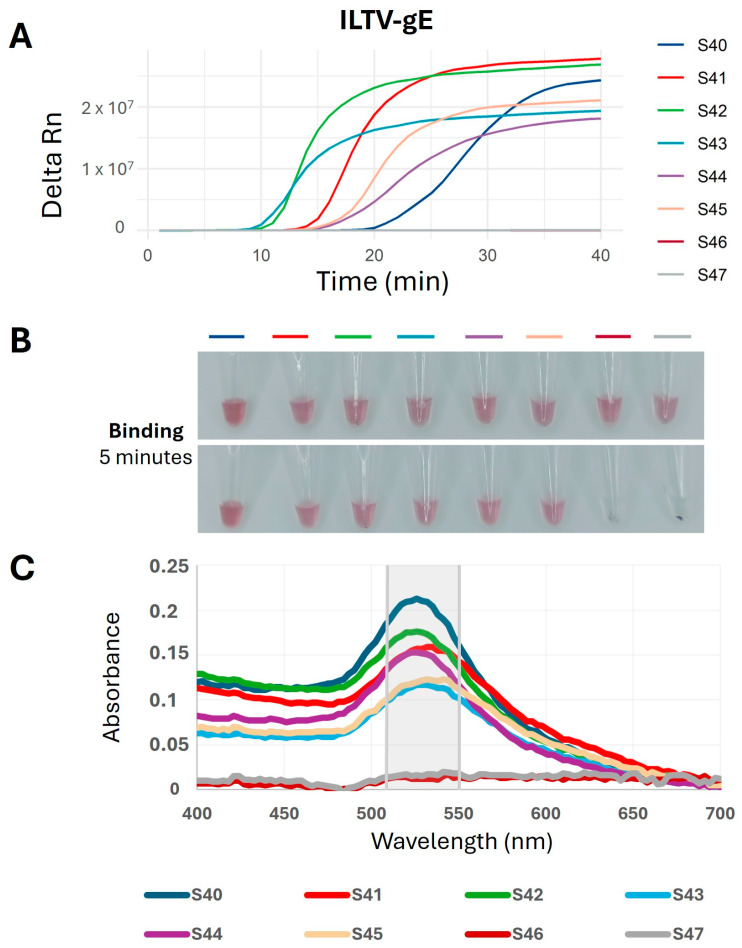
Validation of a two-step LAMP technique coupled with ILTV-gE DNA nanoprobes using crude samples. (**A**) Representative qLAMP amplification plots for ILTV-positive (S40–S45) and ILTV-negative samples (S46–S47) after 40 min of reaction. (**B**) Digital pictures captured after room temperature (RT) incubation of S40 to S47 LAMP products with ILTV-gE DNA nanoprobes, taken at 0 min (upper panel) and 5 min (lower panel). (**C**) VIS-UV spectrum representing the DNA nanoprobes absorbance intensity from 400 nm to 700 nm after 5 min of RT incubation with LAMP products. The region where the maximum absorbance is observed (515–550 nm) is highlighted.

Specific DNA nanoprobes targeting the ILTV-gE amplicon were employed as a colorimetric readout for viral detection coupled with LAMP reaction. The reaction conditions for the color shift of DNA nanoprobes in the presence or absence of the LAMP product were optimized, allowing for clear differentiation between positive (red color) and negative (greyish-blue color after salt addition) results (Figure 6B). Based on our previous optimization [16], we determined that a 1.3:5 ratio of DNA nanoprobes to LAMP product provides an optimal ratio for efficient detection. To achieve clearer results, we determined that an optimal final volume of 15 µL was required for the binding reaction. This involved adding 8.5 µL of binding buffer and incubating the reaction for 5 min at room temperature. This incubation resulted in a readily observable color difference, which was subsequently confirmed by visible-ultraviolet (VIS-UV) spectroscopy (Figure 6B,C).

### 2.5. Limit of Detection (LOD)

**Figure 7:**
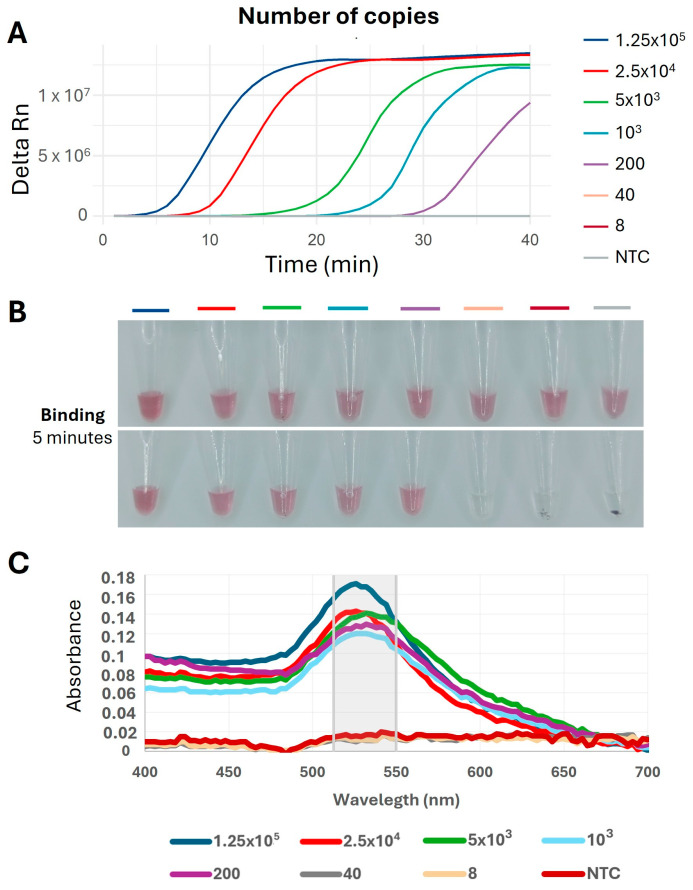
Results of the LOD determination assay using qLAMP coupled with ILTV-gE DNA nanoprobes. (**A**) Amplification plot of qLAMP after 40 min of incubation using serial dilutions of purified TOPO-ILTV-gE plasmid DNA. Each dilution was prepared and analysed in triplicates and amplification plot represents the mean result. (**B**) Digital pictures acquired after room temperature (RT) incubation of the respective LAMP products with ILTV-gE DNA nanoprobes, taken at 0 min (**upper panel**) and 5 min (**lower panel**). (**C**) VIS-UV spectrum representing the DNA nanoprobes absorbance intensity from 400 nm to 700 nm after 5 min of RT incubation with LAMP products. The region where the maximum absorbance is observed (515–550 nm) is highlighted. NTC, non-template control (negative control).

The analytical sensitivity, or limit of detection (LOD), of the system combining LAMP with specific ILTV-gE DNA nanoprobes was determined using serial dilutions of the purified TOPO-ILTV-gE plasmid (Figure 7). Based on the results, the LOD of the ILTV detection system was calculated to be 200 copies per reaction.

### 2.6. Optimized Protocol for Molecular Detection of ILTV

**Figure 8:**
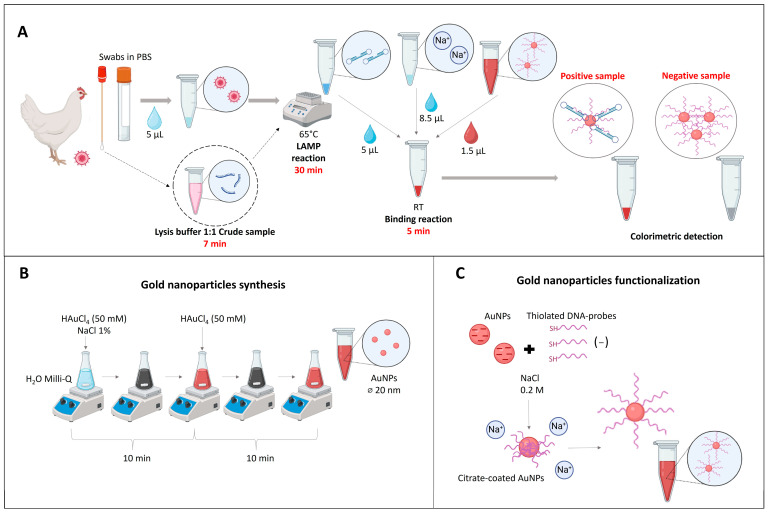
Schematic representation of the experimental procedure. (**A**) LAMP-DNA nanoparticles technique, begins with the sample collection from chickens using respiratory swabs, followed by DNA amplification using LAMP. The detection of amplified LAMP products is then achieved using DNA-functionalized gold nanoparticles, with salt-induced aggregation indicating a positive result. (**B**) Synthesis of 20 nm gold nanoparticles via the Turkevich method. In this process, citrate acts as a reducing and stabilizing agent, conferring a negative charge to the nanoparticles. (**C**) Functionalization of gold nanoparticles (AuNPs) with thiolated DNA probes, although both the AuNPs and the thiolated DNA probes are negatively charged, the addition of salt reduces electrostatic repulsion, allowing for the DNA probes to bind them.

We propose a protocol (Figure 8) for the specific and reliable molecular detection of ILTV, using LAMP combined with DNA nanoprobes. Respiratory tract swabs can be used for amplification, either as crude material resuspended in PBS or after being pretreated with a lysis buffer. To validate this protocol, we compared our results with those obtained using DNA/RNA extracts. The amplification reaction includes 12.5 µL of WarmStart MasterMix with dUTP/UDG, 1.6 µM of primers FIP/BIP, 0.2 µM of primers F3/B3, 0.4 µM of primers LF/BF, and 5 µL of sample, for a total volume of 25 µL. Reactions are conducted at 65 °C for 30 min, and results are visualized by incubating 5 µL of RT-LAMP products with 1.5 µL of DNA nanoprobes (50 µM) in a reaction buffer (2 M NaCl, 80 mM MgCl_2_, and 0.03% Tween-20) for 5 min at room temperature.

## 3. Discussion

Accurate and timely diagnosis of viral respiratory infections in poultry is essential for effective outbreak management and control on poultry farms. However, conventional methods often suffer from limitations in sensitivity, specificity, as well as their reliance on complex laboratory equipment and trained personnel. LAMP-based tests offer a promising alternative, providing rapid and specific detection of viral pathogens in the field, eliminating the need for complex thermal cycling. By targeting specific genome sequences, LAMP can facilitate POC diagnostics, which are crucial for quick decision making in farming environments. The integration of colorimetric-based readouts, such as specific DNA nanoprobes, enhances the ease of use and accessibility of these assays.

For on-farm implementation, our diagnostic system notably simplifies testing by obviating nucleic acid purification using a lysis buffer or direct swab processing. Consequently, the elimination of sample transport to a specialized laboratory is achieved. Moreover, the lack of need for complex thermal cycling offers clear time and cost advantages [12]. Unlike our system, reported diagnostic systems for ILTV and other poultry viruses [6,9,11,14] rely on laboratory-based sample processing and, thus, cannot be considered true POC systems. Notably, without the need for laborious viral genome extraction, our approach achieves ILTV diagnostic sensitivity comparable to standard PCR [17]. Furthermore, this lysis buffer opens up possibilities for multiplexed systems capable of the simultaneous detection of multiple viruses [18].

This study evaluates the performance of LAMP assays targeting the ILTV-gE or -TK genes for a novel on-farm ILTV detection system. While the ILTV-TK gene has been previously assessed [14,19] and is WOAH-recommended for ILTV diagnosis [3], our ILTV-gE gene demonstrated higher sensitivity (100%) compared to the ILTV-TK assay (84%), with both assays exhibiting 100% specificity. To our knowledge, this is the first study to develop LAMP primers targeting the ILTV gE gene, achieving 100% sensitivity and specificity. Thus, the integration of the gE gene target into new on-farm diagnostic systems improves the control and management of the virus directly in poultry production environments.

Additionally, this work presents a LAMP-based assay coupled with colorimetric detection using nanoparticle (AuNP)-based DNA nanoprobes, targeting amplified products with high specificity. This system uses the surface plasmon resonance (SPR) properties of AuNP-based DNA nanoprobes for naked-eye detection, eliminating the need for complex instrumentation. Prior research highlights the potential of AuNP-based systems for pathogen detection, particularly when integrated with LAMP or RT-LAMP and lateral flow biosensors [19,20,21]. Recently, we successfully applied this approach for aMPV detection, achieving high specificity and sensitivity using DNA nanoprobes targeting the aMPV-F gene [16]. By targeting LAMP-amplified viral DNA sequences, this colorimetric readout minimizes false positives from non-specific amplification or primer dimers, thereby enhancing detection accuracy, specificity, and reliability. This improved specificity addresses those limitations of previous one-step LAMP visualization methods, such as those using hydroxynaphthol blue, turbidimetry, or pH-based systems, which often exhibit reduced sensitivity, especially with field samples [12]. In contrast to fluorescence-based detection, the use of DNA nanoprobes obviates the requirement for specialized instrumentation, resulting in substantial cost savings, and further permits the implementation of multiplexed detection systems employing distinct probes.

ILTV viral loads in respiratory samples often exceed 10^3^ genome copies/µL during early infection, enabling reliable detection by most reported systems. While qPCR offers greater sensitivity, isothermal amplification methods like LAMP provide sufficient accuracy for detecting infection at these viral loads, without significant false positives. However, effective early ILTV detection, especially in field settings where sample purity is often a challenge, requires highly sensitive methods. This study focuses on improving the limit of detection (LOD) and optimizing on-farm usability for ILTV diagnostics. Indeed, our combined LAMP-DNA nanoprobe system for ILTV detection achieved an LOD of 200 copies per reaction (40 copies/µL), comparable to previous studies. Similar LODs have been reported for other ILTV targets (100 copies/reaction for the TK gene [13], 200 copies/reaction for a synthetic TK target [14], and 60 copies/µL for the ICP4 gene [22]). However, these methods often lack field validation, a crucial requirement for real-world applicability. Other approaches also present limitations. While a real-time recombinase polymerase amplification (RPA) system reported 100% sensitivity with a 100 copies/reaction LOD using tracheal swabs or lung homogenates [19], its reliance on nucleic acid extraction and fluorescence-based real-time equipment hinders field deployment. Similarly, El-Tholoth et al.’s microfluidic chip-based system targeting the ILTV-polymerase gene [9], though promising for point-of-care (POC) settings, has a high LOD (250 copies/reaction) and reduced sample size, limiting its sensitivity for large-scale diagnostics. The LAMP-DNA nanoprobe system presented in our work addresses these limitations. It significantly improves the LOD for ILTV detection and is optimized for on-farm use, achieving faster detection times, eliminating the need for commercial DNA extraction kits. Moreover, it allows for the visual detection of ILTV within 35 min: 30 min for the LAMP reaction and 5 min for DNA nanoprobe visualization. While a partial lysis buffer slightly increases the assay time, visual detection remains possible within 45 min. These features make this system suitable for point-of-care diagnostics, enabling rapid, simple, cost-effective, and on-site screening for ILTV infection in farm settings.

## 4. Materials and Methods

### 4.1. Clinical Samples and Commercial Vaccine

In this study, a comprehensive analysis was conducted involving a total of 54 chickens obtained from farms in Spain, provided by the Centro de Sanidad Avícola de Cataluña y Aragón (CESAC) and Cobb España. Initially, respiratory tract swab samples from 25 chickens (labelled S1 to S25) and their corresponding total nucleic acid extractions (labelled E1 to E25) were processed. These samples had each been previously validated by qPCR or RT-qPCR at the CESAC. Of these, 15 samples tested positive for ILTV (S1–S15), while 10 (S16–S25) tested positive for other respiratory viruses, either aMPV or IBV. Additionally, seven total nucleic acid extracts (E26 to E32) from other chickens, all of which were positive for ILTV by qPCR at the CESAC, were analyzed. Finally, 22 additional respiratory tract swab samples (S26–S47) were evaluated. Of these, 14 were confirmed positive for ILTV by qPCR at CESAC, while 8 were negative for respiratory viruses.

In total, 79 samples were analyzed, including 47 respiratory tract swabs (44 pharyngeal and 3 choanal swabs) along with a panel of 32 total nucleic acid extractions, hereafter referred to as DNA/RNA extractions (Table 1). All respiratory tract swabs were resuspended in 1 mL of phosphate-buffered saline (PBS) and frozen at −20 °C upon arrival at the laboratory. The panel of DNA/RNA extractions was stored at −80 °C.

Live attenuated ILT vaccine from Poulvac^®^, Salsbury 146 strain (Zoetis Spain S.L., Madrid, Spain) provided in vials with a lyophilized suspension at ≥10^2.5^ EID50 per doses (0.03 mL), was kindly supplied by the company and used as a positive control. The vials were reconstituted in 1 mL of PBS, and 200 µL total nucleic acids was purified using Purelink viral RNA/DNA Mini Kit (Thermo Fisher Scientific, Waltham, MA, USA), following the manufacturer’s recommendations.

### 4.2. LAMP Primers and Oligonucleotide Probes Design

LAMP primers and oligonucleotide probes for the ILTV envelope glycoprotein E (ILTV-gE) and thymidine kinase (ILTV-TK) coding regions were designed with PrimerExplorerV5 (https://primerexplorer.jp/e/, accessed 10 June 2024) using default parameters. This ensured optimal performance by considering GC content (40–65%), secondary structure formation (free energy below −2.5) and melting temperature (Table 2). Primer sets, each comprising four primers (FIP, F3, BIP, and B3), were designed based on target sequence information. The FIP (BIP) primer consists of sequences from the F1c (B1c) and F2 (B2) regions. The F1, F2, and F3 primers are approximately 20 base pairs long and are selected from the target gene, while B1, B2, and B3 are of similar length (20 bp long) and derived from the complementary strand. The regions F1c and F1, as well as B1 and B1c, are complementary to each other. To design the assay, 34 ILTV-gE gene sequences and 127 ILTV-TK gene sequences published in GenBank (https://www.ncbi.nlm.nih.gov/nuccore, accessed 7 June 2024) were compared using Clustal Omega (https://www.ebi.ac.uk/Tools/msa/clustalo/, accessed 7 June 2024). LAMP primers, including loop primers, were selected in the more conserved regions within the gene, as well as in regions with a higher potential for primer sets. Oligonucleotide LAMP primers used in this study were synthesized by Macrogen-Inc. (Seoul, Republic of Korea). The ILTV-gE oligonucleotide probes, used for functionalization of AuNPs, were designed to target a conserved 17-nucleotide sequence within the LAMP product of the viral gE gene (Table 2). Their secondary structure was further evaluated using the RNAfold server from the Vienna RNA Web Service [23]. Oligonucleotide probes with relatively higher Gibbs free energy (ΔG0) values were selected for synthesis. All oligonucleotide probes utilized in this study were synthesized by Sigma-Aldrich (Merck, Darmstadt, Germany) and featured a 5′-thiol modification for enhanced functionality.

### 4.3. LAMP and Quantitative LAMP (qLAMP) Reaction Settings

LAMP assays (end-point and qLAMP) were performed using either crude samples or DNA/RNA extractions with WarmStart^®^ DNA Polymerase or WarmStart^®^ Fluorescent LAMP/RT-LAMP Kit (New England Biolabs, MA, USA), both containing uracil-DNA glycosylase (UDG). To achieve a complete POC diagnostic system, we developed a rapid partial lysis buffer [24] supplemented with Proteinase-K (1U) (NZYTech, Lisbon, Portugal). Swabs from the respiratory tract of chickens, already resuspended in PBS 1X and stored at −20 °C, were used to prepare the reaction by mixing 5 µL of the PBS 1X suspension with an equal volume (1:1) of partial lysis buffer, producing a final volume of 10 µL. This 10 µL mixture was incubated at 56 °C for 5 min for optimal activity of proteinase-K, followed by incubation at 95 °C over 2 min for enzyme inactivation.

The end-point LAMP reaction was performed in a 25 µL volume, consisting of 12.5 µL of WarmStart MasterMix, 1 µL of each primer. The final optimized concentrations were 0.16 µM for internal FIP/BIP primers, 0.1 µM for outer F3/B3 primers, and 0.2 µM for loop LF/LB primers. For DNA/RNA extractions (E1–E32) and vaccines, 2.5 µL of sample was added after 1:1 dilution in Milli-Q water. For crude samples or vaccines pretreated with rapid partial lysis buffer, 5 µL of pretreated sample was added. For crude samples and vaccines without pretreatment, 2.5 µL was used. Milli-Q water was added to adjust the reaction volume to 25 µL. The reaction mixture was prepared at room temperature to allow for UDG activity, followed by incubation at 61–69 °C for 30 min with Bst 2.0 amplification and subsequent enzyme inactivation at 80 °C for 5 min in a Mastercycler^®^ nexus GX2 thermocycler (Eppendorf, Hamburg, Germany). LAMP reactions were analyzed using 2% TAE-agarose gel electrophoresis for visualization.

For qLAMP, 0.5 µL of LAMP fluorescent dye was added to the reaction mix. The fluorescence signal was measured using a QuantStudio™ 5 Real-Time PCR System (Thermo Fisher Scientific), which includes an end-point melt curve step for specificity validation.

To ensure the reproducibility, each purified sample was analyzed in duplicate on separate days by different operators. Crude samples were tested with and without lysis buffer treatment to confirm the robustness of the results.

### 4.4. Gold Nanoparticle Synthesis and Functionalization

The 20 nm AuNPs were synthesized following the Turkevich method [25]. Briefly, 50 mL of Milli-Q water was heated to boiling under gentle agitation in a 50 mL Erlenmeyer flask. Once boiling, the agitation speed was increased to 1100 rpm, and 400 µL of HAuCl4 (50 mM) and 2 mL of NaCl dihydrate (1%) were injected into the center of the vortex created by the stirring magnet. The solution progressed from clear to black and finally to deep red. A 5 cm diameter Petri dish was placed on top of the flask to minimize water evaporation. Heating and refluxing under agitation continued for 10 min to produce 16 nm AuNPs. To achieve the target particle size of 20 nm, an additional 400 µL of 50 mM HAuCl4 solution was injected while keeping the flask on the heat source, and the conditions were maintained for an additional 10 min. The reaction was then stopped and cooled at 23 °C (Figure 8B). The resulting 20 nm AuNPs were centrifuged at 8500 rpm for 15 min using a Minispin (Eppendorf), and the pellet was redispersed in Milli-Q water. This process was repeated until the UV-visible spectrum showed an absorbance of 0.1 at the maximum absorption wavelength of 520 nm.

To functionalize AuNPs with synthetic oligonucleotides, a previously described protocol was followed [16], incorporating an initial dithiothreitol (DTT) nanoprobe reduction and a modified salt-aging process adapted from Hurst et al. [26] (Figure 8C). Following a final rinse to remove unbound probe-oligonucleotides, the resulting DNA nanoprobes were resuspended in 10 mM PBS and stored at 4 °C until use.

### 4.5. Detection by DNA Nanoprobes

To optimize colorimetric detection, 1.5 µL of ILTV-gE or ILTV-TK DNA nanoprobes was incubated with 5 µL of the corresponding end-point LAMP product in a reaction solution buffered with 25 mM Tris at pH 7.5. The reaction buffer was supplemented with binding buffer, containing 2 M NaCl, 80 mM MgCl_2_, and 0.03% Tween-20. The reaction was carried out in a final volume of 15 µL. Nuclease-free water was used as a non-template control (NTC). Colorimetric detection was performed at room temperature for 5 min. Detection parameters were adjusted based on absorbance readings at a wavelength of 400–700 nm, focusing on the maximum absorbance at 540 nm using a NanoDrop 2000 Spectrophotometer (Thermo Fisher Scientific). All binding experiments were performed in duplicate.

### 4.6. Plasmid Constructs

ILTV-gE coding region was amplified using 10 µM of external LAMP primer (F3 and B3) by conventional PCR with AmpliTaq DNA Polymerase (Thermo Fisher Scientific). The PCR product was then cloned using TOPO™ TA Cloning™ Kit and transformed into TOP10 Chemically Competent *E. coli* (Thermo Fisher Scientific). The TOPO-ILTV-gE plasmid was confirmed by automated Sanger sequencing.

### 4.7. Determination of the Limit of Detection (LOD)

To determine the LOD, five-fold dilutions of a plasmid DNA stock at 20 ng/µL were performed. These dilutions, ranging from 250,000 copies to 8 copies, were then used for qLAMP amplification with ILTV-gE primers. Each dilution was tested in triplicate, and the mean values are presented in an amplification plot (Figure 7). The corresponding LAMP products were assayed for DNA nanoprobe detection in duplicate experiments. The plasmid and fragment sizes were used to calculate the number of plasmid copies in each dilution for the LOD calculation. The assay was conducted on two separate days to assess the consistency and reproducibility of the results.

## 5. Conclusions

In conclusion, this study underscores the crucial advancements in diagnosing viral respiratory infections in poultry using LAMP-based assays. The innovative integration of colorimetric readouts with DNA nanoprobes significantly enhances the ease of use and accessibility of these tests. Our research demonstrates that targeting the ILTV-gE gene with LAMP assays achieves 100% sensitivity and specificity, outperforming the ILTV-TK gene assay. This marks the first study to develop primers for the ILTV-gE gene, paving the way for new on-farm diagnostic systems that improve virus control and management in poultry production environments.

The direct processing of swabs in this work eliminates the need for nucleic acid purification, facilitating true point-of-care diagnosis. With this system, we obtained results in only 35 min (30 min for LAMP and 5 min for detection). Optionally, a partial lysis buffer can be used, which slightly increases the total time but still allows for detection in under 45 min, while enabling multiplex amplification of different viruses. These approaches save time and reduce costs associated with sample transportation and laboratory analysis. They provide visible results to the naked eye, without requiring sophisticated equipment, supporting early, rapid, simple, and cost-effective in situ screening for ILTV infections on farms. Although further field trials will be necessary for its validation as a POC system, the high specificity, sensitivity, and ease of use of this test support its immediate applicability on farms.

## Figures and Tables

**Table 1:** Panel of samples.

Number of Chickens	Samples	Sample Type	Result
25	S1–S25 E1–E25	Respiratory tract swabs ^1^ (crude samples) DNA/RNA extractions	15 positive for ILTV 5 positive for IBV 5 positive for aMPV
7	E26–E32	DNA/RNA extractions	7 positive for ILTV
22	S26–S47	Respiratory tract swabs ^1^ (crude samples)	14 positive for ILTV 8 negative for respiratory viruses
Total: 54	Total: 79	Total: 47 Respiratory tract swabs ^1^ (crude samples) 32 DNA/RNA extractions	

^1^ Respiratory tract samples correspond to pharyngeal swabs, except for sample S4 (respiratory tract swab), which was positive for ILTV but lacks specific information on the tract of origin, and samples S16, S17, and S18 (choanal swabs), which were positives for IBV.

**Table 2:** Primer sets for LAMP targeting ILTV-gE and ILTV-TK gene sequences, along with corresponding oligonucleotide probe for colorimetric detection.

Primer	Sequence 5′-3′
ILTV-gE-F3	GGCCATGGAAACTACATCGT
ILTV-gE-B3	GGTCGGTGGGAAGACTRT
ILTV-gE-FIP	CCTCTGACGGCTTGATGGTGTCGCGTTCTTTCGCACGTAGA
ILTV-gE-BIP	CCACGCGCACGTGGAATTACATAGGTGGGGCTGTTGTCG
ILTV-gE-Floop	ATCTCCACCTCGTGCGGTG
ILTV-gE-Bloop	TGCTGCCGTTTCATGAACTCA
ILTV-TK-F3	GAAATACAAACCCTAAAGGCT
ILTV-TK-B3	GGAAACACAAACATGCCG
ILTV-TK-FIP	AGCAATAGTCATCTGAACTTCCGTCCGGAAAACTTGAATGTCG
ILTV-TK-BIP	TTGGGATCTGAACGCTGCTACCTATCTACGAGGAATAAGACA
ILTV-TK-Floop	TACGCGACGAGACGCCT
ILTV-TK-Bloop	CAGCTGCATCCGGACCAGA
ILTV-gE-Probe	TTTTTTTTTTATGTGACCCCTGTTCTTA

## Data Availability

The original contributions presented in this study are included in the article. Further inquiries can be directed to the corresponding authors.

## Abbreviations

The following abbreviations are used in this manuscript:

ILTV	Infectious laryngotracheitis virus
ORFs	Open reading frames
IBV	Infectious bronchitis virus
aMPV	Avian metapneumovirus
LAMP	Loop-mediated isothermal amplification
qPCR	Real time PCR
qLAMP	Quantitative LAMP
POCT	Point-of-care test
AuNPs	Gold nanoparticles
LSPR	Localized surface plasmon resonance
ILTV-gE	ILTV glycoprotein-E
ILTV-TK	ILTV thymidine kinase
NTC	Non-template control
UV	Ultraviolet
LOD	Limit of detection
WHOA	World health organization of animals
ICP4	Infected cell protein 4
aMPV-F	aMPV fusion protein
Bst	*Bacillus stearothermophilus*
